# Impact of thyroid dysfunction on clinical outcome in head and neck cancer: a systematic review and meta-analysis

**DOI:** 10.1186/s12885-025-15004-z

**Published:** 2025-10-17

**Authors:** Sandeepa N. C, Tina S. Roberts, Johan Frank Opperman, Johan Grobbelaar, Muhammed Ajmal

**Affiliations:** 1 Dept. Diagnostic Sciences, College of Dentistry, King Khalid University, Abha, Saudi Arabia; 2 Dept. Craniofacial Biology, Oral Pathology & Radiology, Faculty of Dentistry, University of The Western Cape, Bellville, South Africa; 3 Dept. Craniofacial Biology, Oral Pathology & Radiology, University of the Western Cape, Bellville, South Africa; 4 Division of Otorhinolaryngology, Department of Surgical Sciences, University of Stellenbosch, Stellenbosch, South Africa

**Keywords:** Thyroid dysfunction, Head and neck cancer, Overall survival, Disease-free survival, Progression- free survival

## Abstract

**Background:**

The influence of thyroid dysfunction on the clinical outcomes of head and neck cancer (HNC) patients remains an area of ongoing investigation, with previous studies yielding variable results. Treatments for HNC, particularly radiotherapy, frequently impact thyroid function. This meta-analysis aimed to synthesize the available evidence on the association between thyroid status (dysfunction vs. euthyroid) and survival outcomes in HNC patients.

**Methods:**

A systematic review and meta-analysis were conducted following the Preferred Reporting Items for Systematic Reviews and Meta-Analyses (PRISMA) guidelines. Medline, Web of Science, Cochrane Library, Embase and Scopus were searched (January 2000-October 2024) for studies comparing survival outcomes (primarily Overall Survival) in adult HNC patients with thyroid dysfunction versus euthyroid patients. Two reviewers independently selected studies and extracted relevant data. The risk of bias for included studies was assessed using the Newcastle-Ottawa Scale (NOS) for cohort studies and the Cochrane Risk of Bias 2 (RoB2) tool for randomized controlled trials. Hazard ratios (HRs) with 95% confidence intervals (CIs) comparing survival were pooled using both fixed-effect (common-effect) and random-effects (REML) models. Heterogeneity across studies was assessed using the I² statistic and Cochran’s Q test. Statistical analyses were performed using R with the *meta* package.

**Results:**

Six studies met the inclusion criteria for systematic review. Four of these studies, encompassing 671 participants, reported sufficient data (Hazard Ratios for Overall Survival) for meta-analysis. The fixed-effect model yielded a pooled HR of 0.99 (95% CI: 0.98, 1.00; *p* = 0.0013). However, significant heterogeneity was observed (I² = 81.5%, *p* = 0.0010). Consequently, the random-effects model, deemed more appropriate, yielded a pooled HR of 1.45 (95% CI: 0.66, 3.19; *p* = 0.3601), indicating no statistically significant association between thyroid status and overall survival in HNC patients. The estimated between-study variance (τ²) was 0.53.

**Conclusion:**

This meta-analysis revealed substantial heterogeneity among studies investigating the impact of thyroid status on HNC survival. When accounting for this heterogeneity using a random-effects model, no significant association was found. The findings highlight the need for further research with larger sample sizes, standardized definitions of thyroid dysfunction, consistent reporting of adjusted effect estimates, and exploration of heterogeneity sources.

**Trial registration:**

CRD42024535167.

**Supplementary Information:**

The online version contains supplementary material available at 10.1186/s12885-025-15004-z.

## Introduction

Head and neck cancers (HNC) encompass a diverse group of malignancies arising in the upper aerodigestive tract, posing a significant global health burden [1]. Treatment often involves multimodal approaches including surgery, radiotherapy, and chemotherapy [2]. A common consequence, especially following neck irradiation, is thyroid dysfunction, most frequently hypothyroidism [3]. Thyroid hormones are fundamental to cellular metabolism, growth, and differentiation [4], and preclinical evidence suggests potential roles in cancer biology [5].

However, the clinical impact of thyroid dysfunction (hypothyroidism or hyperthyroidism) on the disease course and survival of HNC patients remains unclear and debated. Some studies suggest an association with poorer outcomes, potentially linked to treatment resistance or altered metabolism, while others report no significant association or even hint at protective effects in certain contexts [6, 7]. This inconsistency across studies necessitates a quantitative synthesis to provide a clearer estimate of the overall association and to guide clinical management.

To address this gap, we structured our review question using the PICO framework. The PICO question guiding this review is: In patients with head and neck cancer (P), does thyroid dysfunction (E), compared to euthyroidism (C), impact clinical outcomes (Overall Survival, Disease-free Survival, Progression-Free Survival) (O)?

This systematic review and meta-analysis aim to synthesise the existing evidence to determine the overall association between thyroid status (dysfunction vs. euthyroidism) and clinical outcomes, primarily overall survival (OS), in patients with head and neck cancer.

## Methods

This systematic review and meta-analysis were conducted and reported following the Preferred Reporting Items for Systematic Reviews and Meta-Analyses (PRISMA) 2020 statement [8]. The review protocol was registered with PROSPERO (ID: CRD42024535167).

### Eligibility criteria

Studies were included based on:

- Population: Patients ≥ 18 years with HNC (specific type/site required).
- Exposure: Assessed thyroid function (T3, T4, TSH) with categorisation (hypothyroid, hyperthyroid) vs. euthyroid, using defined reference ranges.
- Comparison: Euthyroid HNC patients.
- Outcomes: Reported overall survival (OS), disease-free survival (DFS), or progression-free survival (PFS), allowing comparison (HRs).
- Study Design: Retrospective or prospective cohort studies.
- Other: English language published 2000 onwards, ≥ 50 participants.
- Exclusions: No serum hormone levels, no categorisation vs. euthyroid, non-specific HNC, biomarkers/antibodies only, in vitro/animal studies, < 18 years, < 50 participants, case reports/series, reviews, editorials, letters, conference abstracts, books/chapters.

### Information sources

A systematic literature search was conducted across multiple electronic databases: Medline (via PubMed), Embase, Web of Science (Core Collection), Cochrane Library (CENTRAL), and Scopus. The searches aimed to identify relevant studies published from January 1, 2000, up to October 2024. In addition to database searching, the reference lists of all included studies and relevant review articles were manually screened (backward citation searching) to identify potentially eligible publications missed by the electronic searches.

### Search strategy

Comprehensive strategies using MeSH/keywords for HNC, thyroid dysfunction, and outcomes. Limited to English, human, 2000+. The detailed PubMed search strategy is presented in Appendix 1 (supplementary file), and adaptations for additional databases are shown in Appendix 2(supplementary file).

### Study selection

**Fig. 1:**
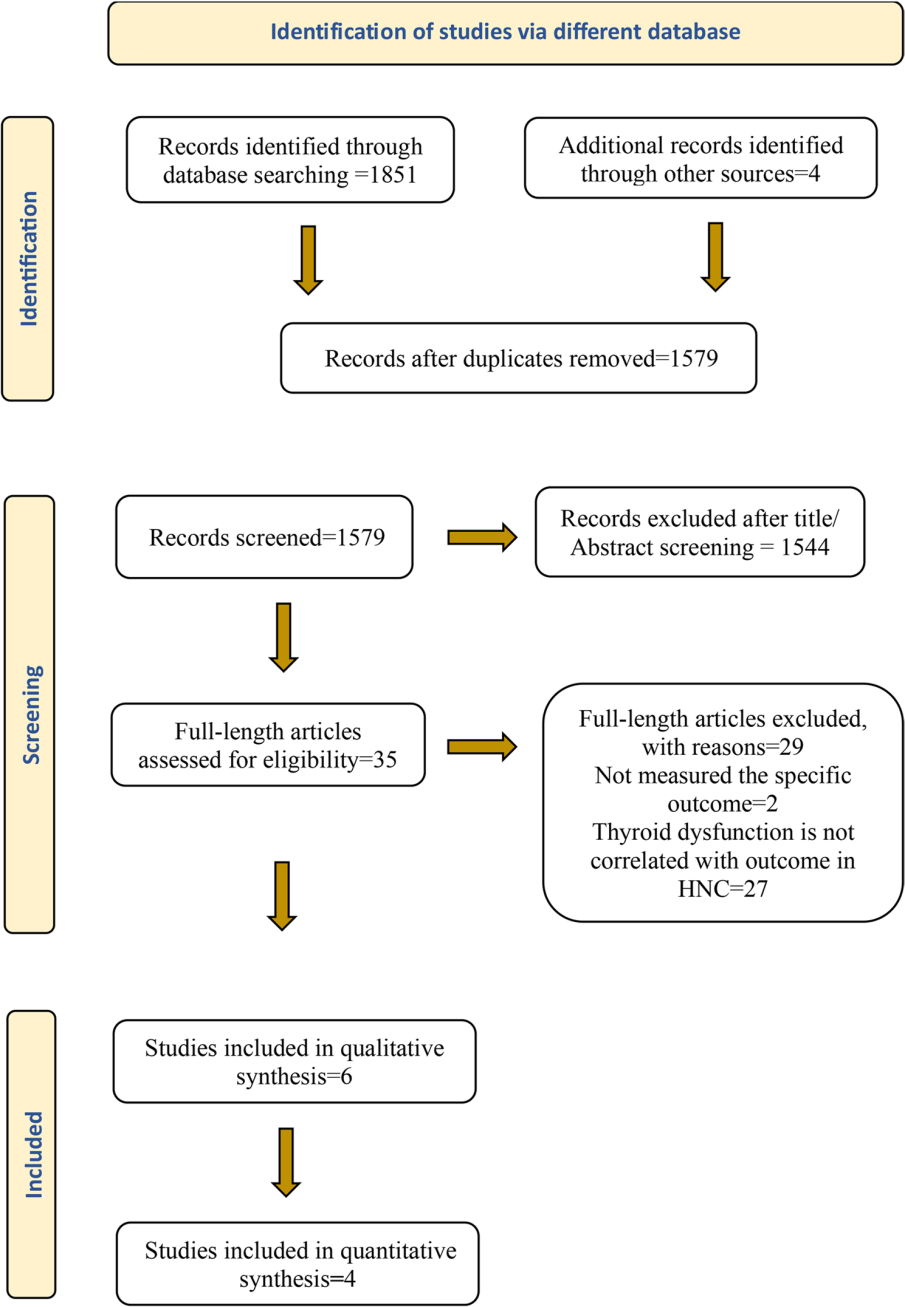
PRISMA flow diagram

Search results from all databases were imported into EndNote, and duplicates were removed. The cleaned library was then exported to an Excel sheet that facilitates blinded and independent screening. Two reviewers (SN, MA) independently screened the titles and abstracts of the retrieved records against the predefined eligibility criteria. Full texts of potentially relevant articles were obtained and assessed independently by the same two reviewers. Any disagreements regarding study inclusion at either the abstract or full-text screening stage were resolved through discussion and consensus, and unresolved conflicts were adjudicated by a third reviewer (TR)who was the arbitrator. The study selection process is documented in the PRISMA flow diagram (Fig. 1).

### Data collection process

A standardised Excel form was used. Two reviewers (SN, MA) independently extracted data. Any discrepancies in extracted data were resolved through discussion or, when necessary, with adjudication by a third reviewer (TR). Where critical data was missing or unclear, the authors of the primary studies were contacted to obtain additional information or clarification.

### Data items

Extracted data included: Study characteristics (author, year, country, design, period, N); Participant characteristics (age, sex, HNC type/site, stage, treatment); Exposure details (assessment method/timing, definitions/ranges for thyroid status, N per group); Outcome data (definitions, follow-up, HR [95% CI] - preferably adjusted, or data to calculate); risk of bias assessment data.

### Risk of bias in individual studies

The methodological quality and risk of bias of included studies were independently assessed by two reviewers (SN, MA).

For cohort studies, the Newcastle-Ottawa Scale (NOS) was utilised [9]. The NOS assesses studies based on three domains: selection of study groups (maximum 4 stars), comparability of groups (maximum 2 stars), and ascertainment of outcome/exposure (maximum 3 stars), for a total possible score of 9 stars. Based on their NOS score, cohort studies were categorised as high quality (≥ 7 stars), moderate quality (5–6 stars), or low quality (≤ 4 stars).

For the randomized controlled trial (RCT), the Cochrane Risk of Bias 2 (RoB2) tool was employed [10]. The RoB2 tool evaluates bias across five domains: bias arising from the randomization process, bias due to deviations from intended interventions, bias due to missing outcome data, bias in measurement of the outcome, and bias in selection of the reported result. An overall risk of bias judgment (Low risk, some concerns, or High risk) was assigned based on the assessments across these domains.

Any disagreements between the two reviewers during the assessment process for either tool were resolved through discussion or consultation with a third reviewer, who is the arbitrator (TR). The results of the risk of bias assessment for all included studies were summarised descriptively and considered during the interpretation of the review findings.

### Statistical analysis

The primary effect measure was the hazard ratio (HR), comparing overall survival in HNC patients with thyroid dysfunction (hypothyroidism or hyperthyroidism, treated as a combined group based on available data from included studies) to that in euthyroid patients. HRs and their corresponding 95% confidence intervals (CIs) were extracted from each study. Log (HR) and standard errors (SE) were used for pooling. Standard errors were calculated from p-values or CIs if not directly reported.

To synthesise the data, both fixed-effect (inverse variance weighted) and random-effects (REML method for τ²) meta-analysis models were employed. The fixed-effect model assumes that all included studies are estimating the same underlying true effect, and any observed differences in study results are due to chance (sampling error). The random-effects model, conversely, assumes that the true effects can vary across studies (i.e., heterogeneity is present beyond chance) and estimates the mean of this distribution of effects.

The choice between these models is critical, especially when their results diverge. In this meta-analysis, substantial and statistically significant heterogeneity was observed among the included studies (Cochran’s Q = 16.22, df = 3, *p* = 0.0010; I² = 81.5%), indicating that the assumption of a single common effect size (as per the fixed-effect model) is likely violated.

Therefore, while both models were run, the random-effects model is considered the primary model for interpreting the overall effect of thyroid dysfunction on survival in HNC patients due to the high level of observed heterogeneity. The random-effects model provides a more conservative and appropriate estimate when study effects are diverse. The fixed-effect model results are also presented, but should be interpreted with caution given the significant heterogeneity. Due to the small number of included studies (k = 4), the estimate of between-study variance (τ²) and the I² statistic should be interpreted with some caution, though the formal test for heterogeneity (Cochran’s Q) supports its presence. Subgroup analyses and formal assessment of publication bias (funnel plot, Egger’s test) were not performed due to the limited number of studies.

Statistical heterogeneity was assessed using:

- Cochran’s Q test (chi-squared test), with *p* < 0.10 considered indicative of significant heterogeneity.
- The I² statistic, quantifying the percentage of total variation across studies attributable to heterogeneity (interpreting < 25% as low, 25–75% as moderate, > 75% as high heterogeneity).
- The H statistics.

All statistical analyses were performed using the R statistical software version 4.3.3 [11] with the meta package version 1.16.0 [12].

## Results

### Study selection

The search identified 1,855 records (databases and manual searching of reference lists), of which 276 duplicates were removed. After screening titles and abstracts, 35 full-text articles were assessed for eligibility. Six studies met all inclusion criteria for qualitative synthesis, and four of these provided sufficient data for quantitative synthesis (meta-analysis). Details of the selection process, including reasons for exclusion, are presented in the PRISMA flow diagram (Fig. 1) and in the Table of Excluded Studies (Supplementary File: Appendix 3).

### Study characteristics

The characteristics of the six studies included in the systematic review are summarised in Tables 1 and 2. All six studies utilised a retrospective design, analysing patient data collected previously. The studies were published between 2006 and 2023. The geographical origin of the studies included India (Patil et al.), China (Weng et al.), Greece (Economopoulou et al.), the USA (Nelson et al.), and Austria (Haas et al., Jank et al.).

**Table 1:** Overall characteristics of the included studies

No	Authors	Study design	Type of HNC & site	Sample size	Age	Gender	Staging of the tumor/Disease status	Participants with thyroid dysfunction	Reference range of TFT/definition used	Outcome measured and their definitions
1	Nelson et al. 2006 Ohio, US [13]	Retrospective	HNSCC. Oropharynx Larynx hypopharynx oral cavity	155	hypothyroid = 57 ± 11 not hypothyroid = 59 ± 11	Male: hypothyroid = 45, not hypothyroid=. 74 Female: hypothyroid = 14, not hypothyroid = 22	II-04 III-38 IV-113	Hypothyroid-59 not hypothyroid-96	Hypothyroidism: TSH > 5.5 mIU/L.	OS: Time until the specific outcome event from the date of onset of radiotherapy.
2	Patil et al. 2018 Mumbai, India [14]	Phase III openlabel, noninferiority randomized study	HNSCC. oral cavity oropharynx larynx hypopharynx head and neck node with unknown primary	300	hypothyroid-43.45 Euthyroid-44.84	Male: 267, female: 33. hypothyroid: male-72 female-12 Euthyroid: male-195 female-21	II-26, IVA-270, IVB-4. Hypothyroid = III-13 IV-71 Euthyroid = III-13 IV-203	Hypothyroid = 84, Euthyroid= 216	Hypothyroidism: TSH > 5 uIU/ml. Hypothyroidism duration: cumulative time in hypothyroid state post-randomization.	OS: time in months from date of randomization to death due to any cause. PFS: as time in months from the date of randomization to any kind of failure or death due to any cause whichever occurred earlier.
3	Economopoulou et al. 2020 Greece [15]	Retrospective	HNSCC. oral cavity oropharynx larynx hypopharynx	89	median age = 65 (range 36–92)	Male-67 Female-22	De novo metastatic: 29, recurrent: 60	thyroiditis = 13 hyperthyroidism = 3, hypothyroidism = 10	Hyperthyroidism: Low TSH with high or normal fT4. Hypothyroidism: Low fT4 (regardless of TSH), or high TSH with low or normal fT4. Reference ranges: TSH 0.27–4.2 µIU/mL, fT4 0.93–1.7 ng/dL.	OS: calculated from the date of initiation of Nivolumab to the date of death
4	Jank et al. 2021 Austria [16]	Retrospective	HNSCC. oral cavity oropharynx hypopharynx larynx	TMA dataset − 121 TCGA dataset-443	59 in TMA	TMA: Male-93, Female-28	I–II 40 III–IV 81	low TSH-5 in TMA	Cut- off for Subclinical hyperthyroidism: TSH < 0.5 µIU/ml. Cut - off for Hypothyroidism: TSH > 5.5 µ IU/mL	OS: time from surgery to the time of death from any cause. DFS: Time from surgery to the histologically confirmed occurrence of recurrence.
5	Weng et al. 2022 China [17]	Retrospective	Nasopharyngeal carcinoma	284. After matching: 216	45.78 ± 11.63. after matching 45.96 ± 12.06	Male-199 female-85 Euthyroid: male-137 female-39. hypothyroid: Male-62, Female-46. After propensity score matching: Male-131, Female-85. euthyroid: Male-69, Female-39. Hypothyroid: Male-62, Female-46	I-11 II-81 III-106 IV-86. after matching: I-8, II-60, III-83, IV-65	Euthyroid-176 Hypothyroid-108. After propensity score matching Euthyroid-108, Hypothyroid-108	Hypothyroidism: TSH > ULN. Subclinical hypothyroidism: TSH > ULN, T3, T4 normal. Clinical hypothyroidism: TSH > ULN, low T3, T4.	OS, DFS
6	Haas et al. 2023 Vienna, Austria [18]	Retrospective	HNSCC Oral cavity Oropharynx Hypopharynx Larynx Multifocal	95	Mean age = 66.2	Male-66 Female-29	Recurrent: 47, Metastatic: 14, Recurrent and metastatic: 34	Baseline thyroid status: Euthyroid-44, Hypothyrid-22 Hyperthyroid-03 Low fT3-26, Immune related thyroid dysfunction: Hypothyroidism (latent or overt): 10 Hyperthyroidism (latent or overt):6	TSH 0.27–4.20µIU/ml, fT4 0.76-1.66ng/dl, fT3 2.15-4.12pg/ml Hypothyroid: TSH > ULN with normal or < LLN fT4 and fT3 ≥ LLN. Hyperthyroid: TSH < LLN, Normal fT4 > ULN. Low fT3: fT3 < LLN. Immune-related hypothyroidism: TSH ↑(> ULN) Immune-related hyperthyroidism :TSH ↓(< LLN)	OS, PFS

Abbreviations: *HNC* Head and neck cancer, *HNSCC* Head and neck squamous cell carcinoma, *TFT* Thyroid function test, *TSH* Thyroid-Stimulating Hormone, *fT4* free thyroxine, *T3* Triiodothyronine, *T4* Thyroxine, *fT3* Free Triiodothyronine, *ULN* Upper limit of normal, *LLN* Lower limit of normal, *OS* Overall Survival, *PFS* Progression-Free Survival, *DFS* Disease-Free Survival, *TMA* Tissue microarray, *TCGA* The Cancer Genome Atlas

**Table 2:** Results/conclusion of the included studies

No	Authors	Type of treatment	Timing of onset of thyroid dysfunction	Outcome measured	Effect on clinical outcome	Conclusion
1	Nelson et al. 2006 Ohio, US [13]	Radiation therapy alone or in combination with chemotherapy and surgery. [Chemotherapy: given during the first and fourth week of radiation therapy. Intravenous fluorouracil (1000 mg/m^2^ per day) and cisplatin (20 mg/m^2^ per day). Radiotherapy: fractionated doses of 1.8 to 2.0 Gy were given, and clinical response was evaluated. after 50–55 Gy administration. Radiation therapy was continued to a total dose of 66–72 Gy for those with an evident response and those with no apparent response were referred for surgery].	22.8 months after treatment initiation (range, 3.8–86.4 months).	OS	Unadjusted: Hypothyroidism associated with better OS HR 0.30(0.17–0.52);(*p* < 0.001). Adjusted: Hypothyroidism associated with better OS, but not significant (HR 0.62, (0.35–1.13) *p* = 0.12). Landmark analysis supported this (*p* = 0.11–0.19).	Hypothyroidism at any point after treatment had improved survival. When the timing until detection of hypothyroidism was considered, the trend continued but didn’t reach statistical significance. Larger prospective studies are needed to confirm the relationship between increased duration of hypothyroidism and outcome.
2	Patil et al. 2018 Mumbai, India [14]	Chemoradiation. [Chemotherapy: Patients were randomised into either the once-in-3-weeks cisplatin (100 mg/m^2^) arm or once-a week cisplatin (30 mg/m^2^) arm. Radiotherapy: 6000 cGy over 6 weeks with daily fraction size of 200 cGy in the adjuvant setting and 7000 cGy over 7 weeks in the radical setting.	For those who developed hypothyroidism, the median time was 6.92 months (interquartile range 4.38–9.94 months), post-completion of chemoradiation.	OS, PFS	On multivariate analysis, for OS, the hypothyroid cohort had a statistically favorable HR of 0.336 (95% CI: 0.175–0.643, *p* = 0.001) and for PFS (HR: 0.579, 95% CI: 0.329–1.022, *p* = 0.059). Higher duration of time spent in a hypothyroid state had a favorable impact on PFS (HR: 0.996, 95% CI: 0.993–0.999, *p* = 0.005) and OS (HR: 0.993, 95% CI: 0.987–0.999, *p* = 0.022). Category 4 (< 40 uIU/ml or > 40 uIU/ml peak TSH value) offered the max advantage for PFS (HR 2.67 (standard error (SE) of HR 0.57, *p* = 0.085) and OS HR (13.42, SE of HR 1.16, *p* = 0.025).	Longer duration and higher TSH (30–40 mIU/ml) may improve oncological outcomes. Further studies are needed to confirm.
3	Economopoulou et al. 2020 Greece [15]	Nivolumab: PD-1 inhibitor median number of cycles administered was 7 (range, 1–40). Those who had progression following Nivolumab received platinum-based/non-platinum-based regimen.	The median time from the first day of nivolumab treatment to irAE onset was 54 days (range: 14–412 days).	OS	Twenty-four patients (27%) developed immune-related adverse events (irAEs), with thyroiditis being the most common event (*N* = 13, 14.6%). Patients with endocrine irAEs (thyroiditis) had better median OS (Log-rank *p* = 0.014) compared to patients without irAEs, while patients with non-endocrine irAEs showed a trend for better OS (Log-rank *p* = 0.074).	Multivariate analysis to identify the prognostic factors for OS included irAE in general. As sample size was small and individual irAE were less, possible correlation between type of iRAE and outcome was not done though an association between endocrine irAE (thyroiditis) with better median OS was observed. Association between hypo/hyperthyroidism and outcome were not done.
4	Jank et al. 2021 Austria [16]	-All patients were treated with surgery followed by postoperative radiotherapy. -Adjuvant radio chemotherapy in some.	TSH levels were evaluated between 2 weeks and 1 day preoperatively.	OS, DFS	Patients with low preoperative thyrotropin levels had a worse prognosis compared to euthyroid patients (5-year overall survival TSH low 20% (95%CI 1–58) vs. TSH norm 58%(95%CI 42–71).Univariable cox model showed significant association of low TSH with OS(HR 2.99, 95%CI 1.01–8.87, *p* = 0.047), but not with DFS (HR 1.54, 95%CI 0.35–6.75, *p* = 0.564).	Patients with subclinical hyperthyroidism had significantly impaired survival (3 times more likely to die) compared to euthyroid patients in univariable cox model. Multivariable models were not calculated due to the low number of observed events.
5	Weng et al. 2022 China [17]	-Radiotherapy alone [IMRT, at a dose of 69–72 Gy] or combined chemoradiotherapy. [Chemotherapy: (i) Cisplatin 30 g/m^2^, iv, days 1–3; 5-fluorouracil 2000 mg/m^2^; every 28 days for 2–3 courses. (ii) Nedaplatin 80 mg/m^2^, iv, day 1, 5-FU 2000 mg/m^2^, ; every 28 days for 2–3 courses].	Hypothyroidism developed 8–99 months after IMRT, with a median time of 46 months.	OS, DFS	Hypothyroidism was noted in 38% of patients. In univariate analysis, the association between hypothyroidism and OS was not statistically significant (*p* = 0.080). Hypothyroidism was associated with better DFS (*p* = 0.002). In multivariate analysis, hypothyroidism was an independent prognostic factor for DFS, with an HR of 0.31 (0.15–0.63) (*p* = 0.001). Thyroxine replacement therapy did not impact survival.	Hypothyroidism is an independent factor influencing the DFS in NPC. Thyroxine replacement therapy did not affect prognosis in hypothyroid patients, and the number of cases needs to be increased to confirm this result.
6	Haas et al. 2023 Vienna, Austria [18]	Pembrolizumab: PD-1 inhibitor Administered at 200 mg or 400 mg every 3 or 6 weeks, respectively. Concurrent chemotherapy with cisplatin or carboplatin, plus 5-fluorouracil, was added for up to 6 cycles in a subset of patients.	Immune-related hyperthyroidism with a median onset of 42 days (range: 20–63 days) after treatment initiation. Immune-related hypothyroidism with a median onset of 95 days (range: 19–155 days).	OS, PFS	The multivariable model showed that baseline Low fT3 [HR: 2.52 (CI: 1.30–4.89); *p* = 0.006] predicted worse OS. While immune-related hyperthyroidism [HR: 0.11 (CI: 0.01–0.89); *p* = 0.038] was related to improved OS. Immune-related hyperthyroidism was also associated with longer PFS [HR: 0.13 (CI: 0.03–0.57); *p* = 0.007].	While baseline Low fT3 predicted worse OS, immune-related hyperthyroidism was related to improved OS and PFS. Baseline fT3 and thyroid monitoring during treatment may help risk-stratify patients. The study had a relatively small sample size.

Abbreviations: *HNC* Head and neck cancer, *HNSCC* Head and neck squamous cell carcinoma, *TFT* Thyroid function test, *TSH* Thyroid-Stimulating Hormone, *fT4* free thyroxine, *T3* Triiodothyronine, *T4* Thyroxine, *fT3* Free Triiodothyronine, *OS* Overall Survival, *PFS* Progression-Free Survival, *DFS* Disease-Free Survival, *irAEs* Immune-Related Adverse Events, *HR* Hazard Ratio, *CI* Confidence Interval, *NPC* Nasopharyngeal Carcinoma, *IMRT* Intensity-Modulated Radiation Therapy

The six studies included in the systematic review represented a total of 1044 participants (Haas *n* = 95, Jank *n* = 121, Nelson *n* = 155, Economopoulou *n* = 89, Weng *n* = 284, and Patil *n* = 300 utilizing a Phase III trial database). Of these, the four studies providing data for the meta-analysis (Haas, Jank, Nelson, Patil) accounted for 671 participants.

Key characteristics varied substantially across the studies:

- Cancer Sites & Stages: The patient populations included those with Head and Neck Squamous Cell Carcinoma (HNSCC) (Haas, Jank, Nelson, Economopoulou), locally advanced Head and Neck Cancer (HNC) (Patil), and specifically Nasopharyngeal Carcinoma (NPC) (Weng). Stages ranged from locally advanced (Patil, Nelson) to recurrent/metastatic (R/M) (Haas, Economopoulou).
- Treatment Context: Treatments varied according to stage and study focus, including primary chemoradiation (Patil, Nelson, Weng), immunotherapy (Pembrolizumab - Haas; Nivolumab - Economopoulou) for R/M disease, and potentially surgery/other modalities depending on the primary treatment setting assessed (Jank, Nelson).
- Thyroid Dysfunction Assessment: There was significant heterogeneity in how thyroid status was defined and assessed. Studies evaluated baseline low fT3 (Haas), preoperative low TSH (Jank), post-treatment hypothyroidism based on elevated TSH (Nelson, Patil, Weng), immune-related thyroiditis/hyperthyroidism (Haas, Economopoulou), and the cumulative *duration* of hypothyroidism (Patil). The timing of assessment (pre- vs. post-treatment) also differed.
- Follow-up: The duration of follow-up was not consistently reported.
- Adjustment for Confounders: Most studies reported using multivariable Cox regression analyses (Haas, Nelson, Economopoulou, Patil, Weng), suggesting adjustment for potential confounders. However, the specific confounders included in the models likely varied between studies. The HR provided by Nelson et al. was explicitly adjusted for the timing of hypothyroidism development. The HR from Patil et al. represented the effect of duration as a continuous variable.

This variability in patient populations, cancer types/stages, treatments, definitions and timing of thyroid assessment, and potential differences in adjusted confounders underscores the clinical heterogeneity present in this body of literature.

### Risk of bias within studies

The included studies’ methodological quality and risk of bias were assessed: five cohort studies using the Newcastle-Ottawa Scale (NOS) and one randomized controlled trial (RCT) using the Cochrane RoB2 tool (supplementary file: Appendix 4). The assessment indicated a generally low risk of bias for cohort studies.

Overall NOS scores ranged from a minimum of 7 to a maximum of 9 out of a possible 9 stars. Based on the pre-defined threshold (≥ 7 stars indicating high quality/low risk of bias), all five cohort studies were rated as high quality. While generally strong, the main variations noted were in the ‘Comparability’ domain (where one study scored 1 star versus 2 stars for the others, potentially related to adjustment for key confounders) and the ‘Adequacy of follow up of cohorts’ (where the same study scored 0 stars versus 1 star for the others). The ascertainment of exposure was consistently rated as high across these studies.

Nelson et al. (7/9): Score reflects concerns about the robustness of adjustment for confounders (as evidenced by loss of significance post-adjustment) and, crucially, inadequate follow-up as suggested by the 0-star rating.

Other Cohort Studies (9/9): These studies generally scored perfectly due to clear definitions for exposure (often lab-based thyroid function tests), objective outcomes, and, importantly, robust methods for ensuring comparability – either through multivariate analysis adjusting for key confounders (like stage, comorbidities, performance status, as inferred for Economopoulou, Haas) or strong design features like propensity score matching (Weng). The Jank et al. score remains slightly ambiguous on comparability as no multivariable models were done on the primary reported cohort, but the 9/9 suggests original assessors were satisfied by other means.

The single RCT was assessed using RoB2 and determined to have “Some concern” regarding the overall risk of bias. This rating stemmed primarily from concerns identified in the domains of ‘Bias Due to Deviations from Intended Interventions’ and ‘Bias in Measurement of the Outcome’. The “Some Concern” is primarily driven by the “open label” design. This inherently raises risks related to deviations from intended interventions (as both patient and clinician behaviour can change with knowledge of treatment) and bias in outcome measurement (if assessors are not blinded, especially for outcomes with subjective components like PFS). While TSH levels are objective, the management and interpretation around achieving/maintaining hypothyroidism and assessing its impact could be influenced in an unblinded setting.

### Results of Synthesis

#### Narrative synthesis

Six studies meeting the inclusion criteria were included in the qualitative synthesis. These studies exhibited considerable variability in their populations, definitions of thyroid dysfunction, treatment contexts, and reported outcomes, contributing to the heterogeneity noted in the subsequent meta-analysis.

- Nelson et al. retrospectively analysed 155 patients with advanced-stage HNSCC treated with radiation +/- chemotherapy/surgery. They observed that patients who developed hypothyroidism (defined as TSH > 5.5 mIU/L) had significantly better survival in unadjusted analysis (*p* < 0.001). However, when adjusting for the timing of hypothyroidism detection using Cox regression, the association was attenuated and no longer statistically significant (HR: 0.62, *p* = 0.12), although still favouring the hypothyroid group. This adjusted HR was used in meta-analysis [13].
- Patil et al. analysed data from a trial database of locally advanced HNC patients post-chemoradiation. They defined hypothyroidism as TSH > 5 uIU/ml. Their analysis focused on the *cumulative duration* spent in a hypothyroid state, finding that a longer duration was significantly associated with improved PFS (HR: 0.996, *p* = 0.005) and OS (HR: 0.993, *p* = 0.022) in multivariate analysis. They also found a potential optimal benefit with peak TSH between 30 and 40 uIU/ml. The HR of 0.99 reported in Table 2 likely represents the effect per unit increase in duration of hypothyroidism, not a direct comparison of hypothyroid status versus euthyroid status at a single point, which complicates its direct pooling with HRs derived from status comparisons [14].
- Economopoulou et al. examined 89 patients with R/M HNSCC treated with nivolumab. They found that the development of *any* immune-related adverse event (irAE), which included thyroiditis in 13 patients (14.6%). Eighty-eight patients were evaluated for the association of irAE with clinical outcome and found significantly longer OS (*p* = 0.004) and post-progression survival (PPS) (*p* = 0.006). However, the study did not report specific survival outcomes comparing patients with thyroiditis versus those without thyroiditis or euthyroid patients. Therefore, it was *excluded from the meta-analysis* as it did not provide an effect estimate specifically for thyroid dysfunction versus a euthyroid comparison group [15].
- Jank et al. evaluated 121 HNSCC patients, assessing preoperative thyrotropin (TSH) levels in a subset of 50. They reported worse 5-year OS in patients with low TSH levels (suggestive of hyperthyroidism or suppressed TSH) compared to euthyroid patients (20% vs. 58%). The HR of 2.99 reported in Table 2 likely reflects this comparison between low TSH and normal TSH groups [16].
- Weng et al. studied 284 nasopharyngeal carcinoma patients post-IMRT. They found hypothyroidism occurred in 38% of patients. After matching propensity scores, survival analysis was done in 216 patients, and hypothyroid patients were associated with significantly *better* disease-free survival (DFS) (*p* = 0.002) and relapse-free survival (RFS) (*p* = 0.008) in multivariate analysis. This study was *excluded from meta-analysis* because it reported on DFS and RFS rather than OS and did not provide Hazard Ratios suitable for pooling [17].
- Haas et al. investigated 95 patients with recurrent/metastatic head and neck squamous cell carcinoma (R/M HNSCC) treated with pembrolizumab. They found that low baseline free T3 (fT3) levels were independently associated with worse overall survival (OS) (HR for low fT3: 2.52, *p* = 0.006), while the development of immune-related hyperthyroidism was associated with improved OS (HR: 0.11, *p* = 0.038) and progression-free survival (PFS). The HR of 3.29 reported in Table 2 likely represents another factor from their multivariate model (platinum-refractory disease) or requires clarification regarding its specific comparison group related to thyroid status used for the meta-analysis [18].

In summary, the findings across the six studies present a mixed picture. Some suggest potential harm from low T3 (Haas) or low TSH (Jank), while others suggest potential benefit from developing hypothyroidism (Nelson, Weng - for DFS/RFS) or immune-related thyroid events (Haas - hyperthyroidism, Economopoulou - any irAE). The study by Patil et al. suggests benefit related to the *duration* of hypothyroidism. Due to differences in reported outcomes (DFS/RFS), lack of specific HRs for thyroid status comparison, or HRs representing continuous duration effects rather than status comparisons, only the studies by Haas et al., Jank et al., Nelson et al., and Patil et al. provided numerical HR data pertaining to OS that were carried forward into the quantitative synthesis, despite the noted heterogeneity in how these HRs were derived or defined.

#### Meta-analysis of overall survival (thyroid dysfunction vs. euthyroid)

Four studies provided HRs for the association between thyroid status (primarily hypothyroidism vs. euthyroid, treated as one group ‘thyroid dysfunction’ for this analysis based on available data) and OS. The characteristics and effect estimate from these studies used in the meta-analysis are shown in Table 3.

**Table 3:** Study data included in the Meta-Analysis for overall survival (Thyroid dysfunction vs. Euthyroid)

S.*N*	Author	HR	*P*-value	SE(log(HR))
1	Haas 2023	3.29	0.002	0.3853
2	Jank 2021	2.99	0.047	0.5514
3	Nelson 2006	0.62	0.120	0.2989
4	Patil 2018	0.99	0.022	0.0031

Pooling these studies using the fixed-effect (common-effect) model yielded a statistically significant pooled HR of 0.99 (95% CI: 0.98, 1.00; *p* = 0.0013).

However, there was evidence of substantial statistical heterogeneity among the studies (Q = 16.22, df = 3, *p* = 0.0010; I² = 81.5% [95% CI: 51.9%, 92.9%]; H = 2.33 [95% CI: 1.44, 3.75]). Given this high heterogeneity, the random-effects model is considered more appropriate.

The pooled HR from the random-effects model was 1.45 (95% CI: 0.66, 3.19), which was not statistically significant (*p* = 0.3601). The estimated between-study variance (τ²) was 0.5348 (SE = 0.7313).

**Fig. 2:**
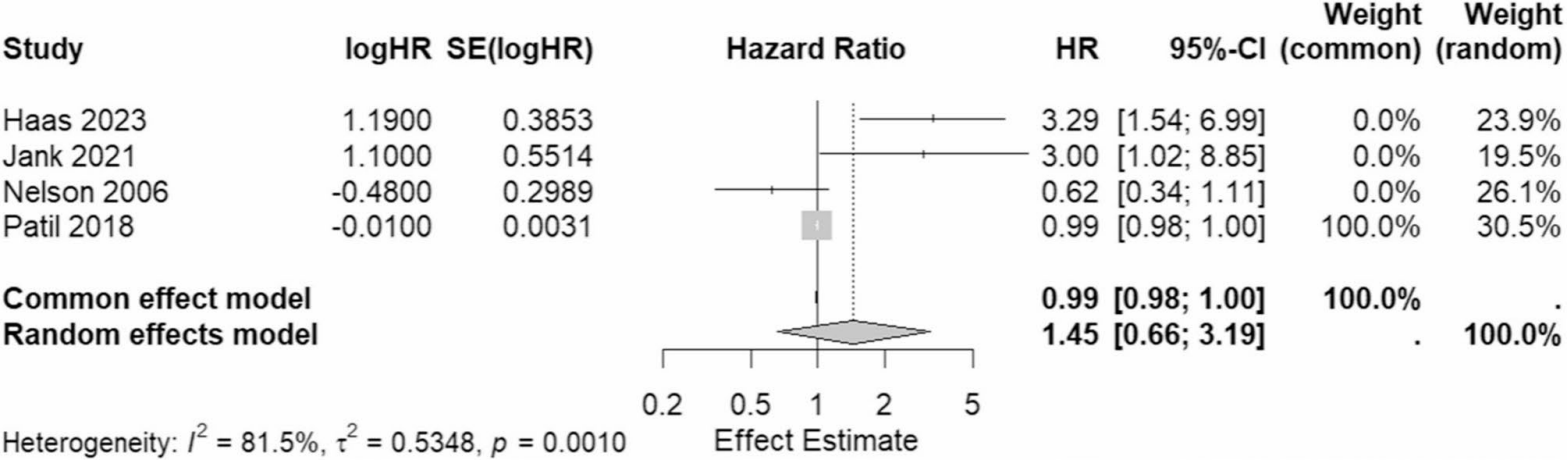
Forest Plot of Meta-Analysis

The results from both models and heterogeneity statistics are summarized in Table 4. The forest plot illustrating individual study HRs and the pooled estimate from both models is presented in Fig. 2.

**Table 4:** Meta-Analysis results for overall survival (Thyroid dysfunction vs. Euthyroid)

	HR	95% CI	Z	*P*-value
Fixed effects model	0.9901	0.9841; 0.9961	−3.21	0.0013
Random effects model	1.4473	0.6557; 3.1945	0.92	0.3601
τ²	0.5348	0.0873; 9.3440		
I²	81.5%	51.9%; 92.9%		
H	2.33	1.44; 3.75		
Test of heterogeneity	Q = 16.22	df = 3		0.0010

Although the fixed-effect model yielded a statistically significant HR of 0.99 (95% CI: 0.984–0.996; *p* = 0.0013), the effect size is very close to 1.0, suggesting minimal clinical relevance. The narrow confidence interval and significance are likely influenced by studies with high precision, particularly Patil et al., which reported a very small standard error.

#### Meta-analysis of secondary outcomes (Disease-Free survival and Progression-Free survival)

Data pertaining to the pre-specified secondary outcomes, Disease-Free Survival (DFS) and Progression-Free Survival (PFS), were extracted where available. However, only two included studies reported results for either DFS or PFS in relation to thyroid dysfunction. Given the limited number of studies providing data on these specific endpoints, a quantitative synthesis via meta-analysis was deemed inappropriate and consequently not performed for DFS and PFS.

Regarding Progression-Free Survival (PFS), Haas et al., (2023) found that immune-related hyperthyroidism was significantly associated with longer PFS (HR: 0.13, *p* = 0.007). Patil et al. (2018) reported several findings related to PFS: comparing the overall hypothyroid cohort to the reference group yielded a Hazard Ratio suggesting potentially longer PFS, though this did not reach statistical significance (HR: 0.579, 95% CI: 0.329–1.022, *P* = 0.059). However, within the hypothyroid group, a longer duration spent in a hypothyroid state was significantly associated with improved PFS (HR: 0.996 per unit increase in duration, 95% CI: 0.993–0.999, *P* = 0.005). Additionally, this study reported a non-significant association for peak TSH values between 30 and 40 uIU/ml (HR 2.67, *P* = 0.085) in relation to PFS.

Regarding Disease-Free Survival (DFS), Weng et al. (2022) found that hypothyroidism was significantly associated with improved DFS based on multivariate analysis (HR 0.31, 95% CI 0.15–0.63, *p* = 0.001). In contrast, Haas et al., (2023), evaluating low thyrotropin (TSH) levels compared to euthyroidism, did not find a statistically significant association with DFS in its univariable analysis (HR 1.54, 95% CI 0.35–6.75, *p* = 0.564).

### Additional analyses

Subgroup analyses (by cancer site, treatment type, definition of hypothyroidism), sensitivity analyses (excluding studies with high risk of bias), and assessment of publication bias were not performed due to the limited number of studies (k = 4) available for the primary meta-analysis.

## Discussion 

### Summary of the main findings

This systematic review and meta-analysis synthesised the available evidence on the association between thyroid status and overall survival in HNC patients. The key finding is the substantial heterogeneity observed among the four studies included in the meta-analysis (I² = 81.5%). This heterogeneity significantly impacts the interpretation of the pooled results.

While the fixed-effect (common-effect) model suggested a statistically significant association (HR 0.99, *p* = 0.0013), this model assumes that all studies estimate the same underlying effect, which is clearly contradicted by the high I² value and significant Q-statistic. The random-effects model, which explicitly accounts for between-study variance, yielded a pooled HR of 1.45 (95% CI: 0.66, 3.19; *p* = 0.36). This estimate is not statistically significant and has a very wide confidence interval, reflecting the considerable uncertainty arising from heterogeneity. Therefore, based on the more appropriate random-effects model, this meta-analysis does not provide evidence for a significant association between thyroid dysfunction (as assessed in these studies) and overall survival in HNC patients.

The statistically significant HR of 0.99 obtained from the fixed-effect model should be interpreted with caution. Despite its apparent significance, the effect size is negligible and likely not clinically meaningful. Moreover, the result may be influenced by one or more studies with extremely small standard errors, leading to high statistical precision. Given the presence of between-study heterogeneity, this estimate may not reflect a biologically plausible or generalizable effect.

We acknowledge that the study by Haas et al. (2023) included patients with metastatic disease treated with immunotherapy, in contrast to other included studies that primarily involved patients receiving radical treatments. Given the relevance of the outcome and alignment with our inclusion criteria, we retained this study in the meta-analysis. To assess its impact, we performed a sensitivity analysis excluding the Hass et al. study. The pooled estimates and conclusion remained unchanged significantly, indicating that its inclusion did not significantly influence the overall results (Supplementary file: Appendix 5).

The pooled estimate was accompanied by substantial heterogeneity (I² = 81.5%). Although we explored potential sources of heterogeneity narratively, formal subgroup or leave-one-out sensitivity analyses were not conducted due to the small number of included studies (k = 4). However, we note that the Patil et al. study, which reported an unusually small standard error, may disproportionately influence the pooled result. This limitation underscores the need for cautious interpretation of the summary estimate and highlights the importance of future studies to characterise the effect better.

Our findings have potential clinical implications for the management of patients with head and neck cancer. Alterations in thyroid function, whether pre-existing or treatment-induced, may influence tumour biology through effects on cellular proliferation, apoptosis, and angiogenesis. Given that thyroid dysfunction can arise from direct tumour effects, surgery, radiotherapy, or systemic therapy, monitoring thyroid hormone levels in HNC patients may aid in early detection of clinically relevant changes and facilitate timely intervention. While the observed associations in our study suggest a potential link between thyroid function and disease progression, causality cannot be inferred, and the clinical relevance of minor hormone fluctuations remains uncertain. Prospective studies are warranted to clarify whether targeted management of thyroid dysfunction could improve outcomes in this patient population.

### Sources of heterogeneity

The substantial heterogeneity observed in the pooled analysis is a major finding and limits definitive conclusions. Several likely sources of variability were identified:

- Differences in Study Populations: Variations in patient demographics (age, sex), baseline comorbidities, distributions of HNC primary sites (larynx vs. oropharynx vs. NPC) and stages, and specific treatment regimens (radiation dose, use of chemotherapy) across studies.
- Differences in Thyroid Function Assessment: Variability in the timing of thyroid assessment (pre-treatment, post-treatment), the specific hormone levels used (TSH alone vs. TSH + fT4), and the cut-off values employed to define euthyroidism, hypothyroidism (subclinical vs. overt), and hyperthyroidism. The studies pooled here likely combined different types or severities of dysfunction.
- Differences in Follow-up Duration: Variations in the length of follow-up could influence observed survival outcomes, particularly for late-onset effects.
- Confounding Factors: Potential differences in unmeasured or inadequately adjusted confounders across studies, such as smoking status, HPV status (especially for oropharyngeal cancer), nutritional status, and performance status, all of which can impact both thyroid function and HNC outcomes. The degree of adjustment in the reported HRs varied.

The included studies were assessed for risk of bias using the Newcastle-Ottawa Scale. As noted in the Results, several studies exhibited methodological limitations, particularly concerning cohort selection, exposure ascertainment, comparability/confounder adjustment, or follow-up adequacy. These limitations may have contributed to the observed heterogeneity and further caution against over-interpreting the pooled estimates.

### Comparison of the studies

Studies examining the relationship between thyroid dysfunction and survival outcomes in HNC have produced mixed results, reflecting the uncertainty observed in this meta-analysis.

For example, Weng et al. (2022) reported a significant association between hypothyroidism and improved disease-free survival (HR 0.31, 95% CI: 0.16–0.59), suggesting a protective effect in nasopharyngeal carcinoma. Patil et al. (2018) found a modest but statistically significant association between the duration of hypothyroidism and improved OS (HR per unit time = 0.993, *p* = 0.022). Conversely, Haas et al. (2023) observed that low baseline free T3 levels were associated with worse overall survival (HR for low fT3: 2.52, *p* = 0.006), and immune-related hyperthyroidism was associated with improved OS (HR: 0.11, *p* = 0.038) and progression-free survival (PFS).

These inconsistencies may reflect true biological variation or differences in study design, thyroid dysfunction classification, or population risk. Our findings align with this broader inconsistency: while individual studies suggest both positive and negative associations, the pooled evidence—when analyzed appropriately using a random-effects model—does not support a consistent or statistically significant relationship.

### Strength and limitations

#### Strengths

- Adherence to PRISMA guidelines.
- Comprehensive database search with manual citation tracking.
- Independent, duplicate screening and data extraction.
- Use of validated tools (RoB2 and NOS) for quality assessment.
- Application of random-effects meta-analysis to account for between-study variance.

#### Limitations

- Small Number of Studies: Only four studies could be included in the meta-analysis, limiting statistical power and the robustness of the findings.
- High Heterogeneity: The substantial unexplained heterogeneity (I²=81.5%) is the primary limitation, making the pooled random effects estimate uncertain and potentially misleading if interpreted as a single true effect.
- Inability to Explore Heterogeneity: Due to the small number of studies, planned subgroup analyses (by type of dysfunction, cancer site, treatment) and meta-regression to investigate sources of heterogeneity could not be performed.
- Potential for Publication Bias: Although not formally assessed due to the small k, publication bias cannot be ruled out. Studies finding null or unexpected results may be less likely to be published.
- Variable Data Quality: Reliance on reported HRs, which may have been adjusted for different confounders across studies, introduces variability. The definition and grouping of “thyroid dysfunction” likely varied. The reliability of the meta-analysis is impacted by this variable data quality, as inconsistent HR adjustments and definitions of thyroid dysfunction limit direct study comparison. The outlier precision reported for the Patil et al. 2018 study adds another layer of uncertainty, potentially skewing the overall estimate if its data or calculation is atypical.
- Limited Evidence Base for Secondary Outcomes: The analysis of secondary outcomes (Disease-Free Survival and Progression-Free Survival) was constrained by a scarcity of data, with only two studies reporting on each endpoint. This limited evidence base prevents robust conclusions regarding the association between thyroid dysfunction and disease recurrence or progression and highlights a significant gap in the current literature.
- Inability to Synthesise or Assess Consistency for Secondary Outcomes: Due to the availability of only two studies for DFS and PFS respectively, a quantitative synthesis (meta-analysis) could not be performed. Consequently, it was not possible to derive a pooled estimate of effect, assess the consistency of findings, or explore potential heterogeneity for these important secondary clinical endpoints.

### Future directions: implications for practice and research

This meta-analysis found substantial heterogeneity among studies examining the association between thyroid status and overall survival in HNC patients. When this heterogeneity was accounted for using a random-effects model, no statistically significant association was detected. The current evidence is insufficient to conclude whether thyroid dysfunction impacts survival in HNC patients due to the limited number of studies and high variability in their results and methodologies. Further research is clearly warranted.

Future research should focus on:

- Large, well-designed prospective cohort studies.
- Standardised, clearly defined criteria for classifying thyroid status (e.g., distinguishing subclinical vs. overt hypothyroidism, timing of assessment relative to treatment).
- Detailed reporting of patient characteristics, HNC specifics (site, stage, HPV status), and treatment details.
- Consistent and comprehensive adjustment for key potential confounders.
- Sufficient follow-up duration.
- Reporting of results (e.g., adjusted HRs with CIs or SEs) in a format amenable to meta-analysis, potentially stratified by important subgroups.
- Collaborative efforts to pool individual patient data could also help overcome limitations of study-level meta-analysis and allow for more detailed exploration of heterogeneity.

## Supplementary Information

Supplementary Material 1.

## Data Availability

The datasets used and/or analysed during the current study are available from the corresponding author on reasonable request.

## Abbreviations

HNC: Head and neck cancer
HNSCC: Head and neck squamous cell carcinoma
TFT: Thyroid function test
TSH: Thyroid-stimulating hormone
fT4: Free thyroxine
T3: Triiodothyronine
T4: Thyroxine
fT3: Free Triiodothyronine
OS: Overall survival
PFS: Progression-free survival
DFS: Disease-free survival
irAEs: Immune-related adverse events
HR: Hazard ratio
CI: Confidence interval
SE: Standard error
NPC: Nasopharyngeal carcinoma
IMRT: Intensity-modulated radiation therapy
R/M: Recurrent /Metastatic
