# Supplementary material for: Impact of thyroid dysfunction on clinical outcome in head and neck cancer: a systematic review and meta-analysis
Source: BMC Cancer. 2025 Oct 17;25:1605. doi: 10.1186/s12885-025-15004-z (PMC12535142; doi:10.1186/s12885-025-15004-z)
Supplement: Supplementary file 1 — Supplementary Material 1. [file 12885_2025_15004_MOESM1_ESM.docx]

**Appendix 1: Search strategy of PubMed**

| Database | Search strategy |
| --- | --- |
| **PubMed** | ((Thyroid Dysfunction OR Hypothyroidism OR Hyperthyroidism) AND (Head and Neck Cancer OR Nasopharyngeal carcinoma OR Head and Neck Squamous cell carcinoma OR laryngeal cancer OR Oral cancer OR Salivary gland cancer OR Oropharyngeal cancer OR Metastatic head and neck squamous cell carcinoma) AND (Overall Survival OR Disease- Free Survival OR Progression-Free Survival OR Outcome OR Treatment response OR Disease outcome OR Survival) |

**Appendix 2: Search strategy for additional databases**

| Database | Search strategy |
| --- | --- |
| **Web of Science** | ((TS=("thyroid dysfunction" OR "hypothyroidism" OR "hyperthyroidism" )) AND TS=("Head and Neck Cancer" OR "Nasopharyngeal carcinoma" OR "Head and Neck Squamous cell carcinoma" OR "laryngeal cancer" OR "Oral cancer" OR "Salivary gland cancer "OR" Oropharyngeal cancer" OR "Metastatic head and neck squamous cell carcinoma")) AND TS=("Overall Survival" OR "Disease- Free Survival" OR "Progression-Free Survival "OR "Outcome" OR "Treatment response "OR "Disease outcome "OR "Survival)") |
| **Cochrane** | "hypothyroidism " OR "Hyperthyroidism" OR "thyroid dysfunction" in Title Abstract Keyword AND "head and neck cancer" OR "head and neck squamous cell carcinoma" in Title Abstract Keyword AND "survival" OR "outcome" OR "Overall Survival" OR "Disease- Free Survival" OR "Progression-Free Survival" in Title Abstract Keyword |
| **Scopus** | ( TITLE-ABS-KEY ( "Head and neck cancer " OR "head and neck squamous cell carcinoma" OR "Nasopharyngeal carcinoma" OR " laryngeal cancer" OR "Oral cancer" OR "Oropharyngeal cancer" OR "Metastatic head and neck squamous cell carcinoma" ) AND TITLE-ABS-KEY ( "Thyroid dysfunction" OR "hypothyroidism" OR "hyperthyroidism" ) AND TITLE-ABS-KEY ( "Overall survival" OR "Disease- Free Survival" OR "Progression-Free Survival" OR "Outcome" OR "Treatment response" OR "Disease outcome" OR "Survival" ) ) |
| **Embase** | (thyroid AND dysfunction OR hypothyroidism OR hyperthyroidism) AND (head AND neck AND cancer OR head) AND neck AND squamous AND cell AND carcinoma AND ('overall survival'/exp OR 'overall survival' OR (overall AND ('survival'/exp OR survival)) OR 'disease- free survival'/exp OR 'disease- free survival' OR (('disease-'/exp OR disease-) AND free AND ('survival'/exp OR survival)) OR 'progression free survival'/exp OR 'progression free survival') |

**Appendix 3: List of excluded studies with reasons**

| **No** | **Author** | **Title** | **Reason for exclusion** |
| --- | --- | --- | --- |
|  | Hilly O et al 2011[1] | Thyroid gland involvement in advanced laryngeal cancer: Association with clinical and pathologic characteristics | Thyroid gland involvement in surgery is correlated with outcome; no correlation is done with thyroid dysfunction and outcome |
|  | Nayak S P et al 2013[2] | Mechanism of Thyroid Gland Invasion in Laryngeal Cancer and Indications for Thyroidectomy | Thyroid gland invasion is correlated with the outcome (which is diagnosed by histopathology). |
|  | Cao CN et al 2016[3] | Nasopharyngeal carcinoma with intracranial extension in the era of intensity-modulated radiotherapy: case–control study using propensity score matching method | Nasopharyngeal carcinoma patients with intra cranial extension / without intra cranial extension are correlated with outcome. Thyroid dysfunction is not correlated with the outcome. |
|  | Hori R et al 2019[4] | Real-world outcomes and prognostic factors in patients receiving nivolumab therapy for recurrent or metastatic head and neck carcinoma | Thyroid dysfunction is not separately correlated with the outcome. It is combined as treatment related adverse events and correlated with outcome. |
|  | Lei M et al 2019 [5] | Evaluation of the impact of thyroiditis development in patients receiving immunotherapy with programmed cell death-1 inhibitors | Thyroiditis is correlated with the outcome, but it is done in a group of tumors. |
|  | Matsuo M 2019[6] | Relationship between immune-related adverse events and the long-term outcomes in recurrent/metastatic head and neck squamous cell carcinoma treated with nivolumab | Endocrine events correlate with the outcome, which is a combination of thyroid disease, hypophysis and hyperglycemia. |
|  | McGuire JK et al 2019[7] | Does Thyroid Gland Preserving Total Laryngectomy Affect Oncological Control in Laryngeal Carcinoma? | Thyroid preserving /sacrificing group (based on the operative procedures) are correlated with outcome; thyroid dysfunction is not correlated. |
|  | Okamoto I  2019[8] | Efficacy and safety of nivolumab in 100 patients with recurrent or metastatic head and neck cancer–a retrospective multicenter study | Immune related adverse events (IrAEs) are correlated with outcomes in which thyroid dysfunction is a part. |
|  | Philips R 2019[9] | The high stakes of head and neck surgery following radiation and chemotherapy – An assessment of complications and survival | Medical complications and outcomes are correlated (thyroid dysfunction are grouped under medical complications) |
|  | Rogado J et al 2019 [10] | Immune-related adverse events predict the therapeutic efficacy of anti-PD-1 antibodies in cancer patients | IrAEs are correlated with the outcome in group of tumors, Thyroid dysfunction is not separately correlated in HNC. |
|  | Sakakida T et al  2019[11] | Clinical features of immune-related thyroid dysfunction and its association with outcomes in patients with advanced malignancies treated by PD-1 blockade | Thyroid dysfunction is correlated with the outcome in a group of tumors. It is not done separately in HNC. |
|  | Ueki Y 2019[12] | Predicting the treatment outcome of nivolumab in recurrent or metastatic head and neck squamous cell carcinoma: prognostic value of combined performance status and modified Glasgow prognostic score | IrAEs are correlated with the outcome in which thyroid dysfunction is a part. |
|  | Schweizer C et al 2020[13] | Prospective evaluation of the prognostic value of immune-related adverse events in patients with non-melanoma solid tumor treated with PD-1/PD-L1 inhibitors alone and in combination with radiotherapy | IrAEs are correlated with outcome in group of tumors. Thyroid dysfunctions are grouped under the iRAEs. |
|  | Yen CJ 2020[14] | Two-year follow-up of a randomized phase III clinical trial of nivolumab vs. the investigator's choice of therapy in the Asian population for recurrent or metastatic squamous cell carcinoma of the head and neck | Treatment related adverse events, in general are correlated with outcome. |
|  | Aslıer M et al 2021[15] | The prognostic value of thyroid gland invasion in locally advanced laryngeal cancers | Thyroid gland invasion(histological) is correlated with outcome. Hypothyroidism/hyperthyroidism are evaluated though not correlated with outcome. |
|  | Inaba H et al 2021[16] | Distinct clinical features and prognosis between persistent and temporary thyroid dysfunctions by immune-checkpoint inhibitors | Thyroid dysfunction is correlated with the outcome in a group of tumors. It is not separately correlated in HNC. |
|  | Lima Ferreira J 2021[17] | Improved survival in patients with thyroid function test abnormalities secondary to immune-checkpoint inhibitors | Thyroid function test abnormalities and Outcome are correlated in a group of tumors; not separately done in HNC. |
|  | Rubino R 2021[18] | Endocrine-related adverse events in a large series of cancer patients treated with anti-PD1 therapy | Endocrine events are correlated with outcome, in combination of tumours. |
|  | Kuusisalo S et al 2022[19] | Association of Rare Immune-Related Adverse Events to Survival in Advanced Cancer Patients Treated with Immune Checkpoint Inhibitors: A Real-World Single-Center Cohort Study | Thyroid dysfunction is not separately correlated with the outcome and group of tumors are included. |
|  | Yoshikawa Y et al 2022 [20] | Prevalence of immune-related adverse events and anti-tumor efficacy following immune checkpoint inhibitor therapy in Japanese patients with various solid tumors | IrAEs in general are correlated in group of tumors with outcome |
|  | Chen ZH et al 2023 [21] | Thyroid dysfunction in Chinese  nasopharyngeal carcinoma after anti‑PD‑1  therapy and its association with treatment  response | Indirect measure of survival did not measure the specific outcome. Association between thyroid dysfunction and treatment response is evaluated. |
|  | Lee HJ et al 2023 [22] | Permanent hypothyroidism following immune check point inhibitor induced thyroiditis may be associated with improved survival: result of an exploratory study | Correlated thyroid dysfunction with outcome in group of tumors. |
|  | Kotevski DP et al  2023[23] | Empirical comparison of routinely collected electronic health record data for head and neck cancer-specific survival in machine-learnt prognostic models | The outcome measured is different which is cancer specific survival |
|  | Wahli M N et al  2023[24] | The role of immune checkpoint inhibitors in clinical practice: an analysis of the treatment patterns, survival and toxicity rates by sex | IrAEs are correlated with outcome in group of tumors. |
|  | Okada T et al 2023[25] | Effects of Pembrolizumab in Recurrent/Metastatic Squamous Cell Head and Neck Carcinoma: A Multicenter Retrospective Study | IrAEs are correlated with outcome which included hypothyroidism. |
|  | Alnemri A E et al 2024  [26] | Predictive capacity of immune-related adverse events and cytokine profiling in neoadjuvant immune checkpoint inhibitor trials for head and neck squamous cell carcinoma | Immune related adverse events which included thyroid dysfunction are correlated with the outcome. Individually thyroid dysfunction and outcome are not correlated. |
|  | Ariizumi Y et al 2024[27] | Extent of thyroidectomy and paratracheal lymph node dissection in total pharyngolaryngectomy for pyriform sinus cancer, and recurrence, survival, and postoperative hypoparathyroidism: A multicenter retrospective study | Thyroidectomy is correlated with outcome; thyroid dysfunction is evaluated but not correlated with outcome. |
| 28 | Kennedy OJ et al 2024 [28] | Thyroid dysfunction after immune checkpoint inhibitors in a single centre  UK pan-cancer cohort: A retrospective study | Correlated thyroid dysfunction with outcome in group of tumors in which head and neck cancers are part of it. |
| 29 | Thapa A et al 2024  [29] | The UK Divide: Does Having a Pembrolizumab–Chemotherapy Option in Head and Neck Cancer Matter? Real-world Experience of First-line Palliative Pembrolizumab Monotherapy and Pembrolizumab–Chemotherapy Combination in Scotland | IrAEs are combined and correlated with outcome. |

**References**

1. Hilly O, Raz R, Vaisbuch Y, Strenov Y, Segal K, Koren R, Shvero J. Thyroid gland involvement in advanced laryngeal cancer: association with clinical and pathologic characteristics. Head Neck. 2012 Nov;34(11):1586-90. doi: 10.1002/hed.21972.
2. Nayak SP, Singh V, Dam A, Bhowmik A, Jadhav TS, Ashraf M, Shah RK, Biswas J. Mechanism of thyroid gland invasion in laryngeal cancer and indications for thyroidectomy. Indian J Otolaryngol Head Neck Surg. 2013 Jul;65(Suppl 1):69-73. doi: 10.1007/s12070-012-0530-9.
3. Cao CN, Luo JW, Gao L, Xu GZ, Yi JL, Huang XD, Li SY, Xiao JP, Zhang Z. Nasopharyngeal carcinoma with intracranial extension in the era of intensity-modulated radiotherapy: case-control study using propensity score matching method. Eur Arch Otorhinolaryngol. 2016 Aug;273(8):2209-15. doi: 10.1007/s00405-015-3749-8
4. Hori R, Shinohara S, Kojima T, Kagoshima H, Kitamura M, Tateya I, Tamaki H, Kumabe Y, Asato R, Harada H, Kitani Y, Tsujimura T, Honda K, Ichimaru K, Omori K. Real-World Outcomes and Prognostic Factors in Patients Receiving Nivolumab Therapy for Recurrent or Metastatic Head and Neck Carcinoma. Cancers (Basel). 2019 Sep 6;11(9):1317. doi: 10.3390/cancers11091317.
5. Lei M, Michael A, Patel S, Wang D. Evaluation of the impact of thyroiditis development in patients receiving immunotherapy with programmed cell death-1 inhibitors. J Oncol Pharm Pract. 2019 Sep;25(6):1402-1411. doi: 10.1177/1078155219829813. Epub 2019 Feb 19
6. Matsuo M, Yasumatsu R, Masuda M, Toh S, Wakasaki T, Hashimoto K, Taura M, Uchi R, Nakagawa T. Relationship between immune-related adverse events and the long-term outcomes in recurrent/metastatic head and neck squamous cell carcinoma treated with nivolumab. Oral Oncol. 2020 Feb;101:104525. doi: 10.1016/j.oraloncology.2019.104525.
7. McGuire JK, Viljoen G, Rocke J, Fitzpatrick S, Dalvie S, Fagan JJ. Does Thyroid Gland Preserving Total Laryngectomy Affect Oncological Control in Laryngeal Carcinoma? Laryngoscope. 2020 Jun;130(6):1465-1469. doi: 10.1002/lary.28235.
8. Okamoto I, Sato H, Kondo T, Koyama N, Fushimi C, Okada T, Miura K, Matsuki T, Yamashita T, Omura G, Tsukahara K. Efficacy and safety of nivolumab in 100 patients with recurrent or metastatic head and neck cancer - a retrospective multicentre study. Acta Otolaryngol. 2019 Oct;139(10):918-925. doi: 10.1080/00016489.2019.1648867.
9. Philips R, Seim N, Marcinow A, Rocco J, Agrawal A, Ozer E, Carrau R, Kang S, Old M. The high stakes of head and neck surgery following radiation and chemotherapy - An assessment of complications and survival. Oral Oncol. 2019 Jul;94:14-20. doi: 10.1016/j.oraloncology.2019.05.004.
10. Rogado J, Sánchez-Torres JM, Romero-Laorden N, Ballesteros AI, Pacheco-Barcia V, Ramos-Leví A, Arranz R, Lorenzo A, Gullón P, Donnay O, Adrados M, Costas P, Aspa J, Alfranca A, Mondéjar R, Colomer R. Immune-related adverse events predict the therapeutic efficacy of anti-PD-1 antibodies in cancer patients. Eur J Cancer. 2019 Mar;109:21-27. doi: 10.1016/j.ejca.2018.10.014.
11. Sakakida T, Ishikawa T, Uchino J, Chihara Y, Komori S, Asai J, Narukawa T, Arai A, Kobayashi T, Tsunezuka H, Kosuga T, Konishi H, Hongo F, Inoue M, Hirano S, Ukimura O, Itoh Y, Taguchi T, Takayama K. Clinical features of immune-related thyroid dysfunction and its association with outcomes in patients with advanced malignancies treated by PD-1 blockade. Oncol Lett. 2019 Aug;18(2):2140-2147. doi: 10.3892/ol.2019.10466
12. Ueki Y, Takahashi T, Ota H, Shodo R, Yamazaki K, Horii A. Predicting the treatment outcome of nivolumab in recurrent or metastatic head and neck squamous cell carcinoma: prognostic value of combined performance status and modified Glasgow prognostic score. Eur Arch Otorhinolaryngol. 2020 Aug;277(8):2341-2347. doi: 10.1007/s00405-020-05945-5. E
13. Schweizer C, Schubert P, Rutzner S, Eckstein M, Haderlein M, Lettmaier S, Semrau S, Gostian AO, Frey B, Gaipl US, Zhou JG, Fietkau R, Hecht M. Prospective evaluation of the prognostic value of immune-related adverse events in patients with non-melanoma solid tumour treated with PD-1/PD-L1 inhibitors alone and in combination with radiotherapy. Eur J Cancer. 2020 Nov;140:55-62. doi: 10.1016/j.ejca.2020.09.001.
14. Yen CJ, Kiyota N, Hanai N, Takahashi S, Yokota T, Iwae S, Shimizu Y, Hong RL, Goto M, Kang JH, Li WSK, Ferris RL, Gillison M, Endo T, Jayaprakash V, Tahara M. Two-year follow-up of a randomized phase III clinical trial of nivolumab vs. the investigator's choice of therapy in the Asian population for recurrent or metastatic squamous cell carcinoma of the head and neck (CheckMate 141). Head Neck. 2020 Oct;42(10):2852-2862. doi: 10.1002/hed.26331
15. Aslıer M, Uçurum BE, Kaya HC, Coskun H. The prognostic value of thyroid gland invasion in locally advanced laryngeal cancers. Acta Otolaryngol. 2021 Sep;141(9):865-872. doi: 10.1080/00016489.2021.1962013.
16. Inaba H, Ariyasu H, Iwakura H, Kurimoto C, Takeshima K, Morita S, Furuta H, Hotomi M, Akamizu T. Distinct clinical features and prognosis between persistent and temporary thyroid dysfunctions by immune-checkpoint inhibitors. Endocr J. 2021 Feb 28;68(2):231-241. doi: 10.1507/endocrj.EJ20-0371. Epub 2020 Oct 3.
17. Lima Ferreira J, Costa C, Marques B, Castro S, Victor M, Oliveira J, Santos AP, Sampaio IL, Duarte H, Marques AP, Torres I. Improved survival in patients with thyroid function test abnormalities secondary to immune-checkpoint inhibitors. Cancer Immunol Immunother. 2021 Feb;70(2):299-309. doi: 10.1007/s00262-020-02664-y.
18. Rubino R, Marini A, Roviello G, Presotto EM, Desideri I, Ciardetti I, Brugia M, Pimpinelli N, Antonuzzo L, Mini E, Livi L, Maggi M, Peri A. Endocrine-related adverse events in a large series of cancer patients treated with anti-PD1 therapy. Endocrine. 2021 Oct;74(1):172-179. doi: 10.1007/s12020-021-02750-w.
19. Kuusisalo S, Koivunen JP, Iivanainen S. Association of Rare Immune-Related Adverse Events to Survival in Advanced Cancer Patients Treated with Immune Checkpoint Inhibitors: A Real-World Single-Center Cohort Study. Cancers (Basel). 2022 May 3;14(9):2276. doi: 10.3390/cancers14092276.
20. Yoshikawa Y, Imamura M, Yamauchi M, Hayes CN, Aikata H, Okamoto W, Miyata Y, Okada M, Hattori N, Sugiyama K, Yoshioka Y, Toratani S, Takechi M, Ichinohe T, Ueda T, Takeno S, Kobayashi T, Ohdan H, Teishima J, Hide M, Nagata Y, Kudo Y, Iida K, Chayama K. Prevalence of immune-related adverse events and anti-tumor efficacy following immune checkpoint inhibitor therapy in Japanese patients with various solid tumors. BMC Cancer. 2022 Nov 29;22(1):1232. doi: 10.1186/s12885-022-10327-7.
21. Chen ZH, Zheng WH, Wu CF, Kou J, Yang XL, Lin L, Lv JW, Sun Y, Zhou GQ. Thyroid dysfunction in Chinese nasopharyngeal carcinoma after anti-PD-1 therapy and its association with treatment response. BMC Med. 2023 Jan 16;21(1):18. doi: 10.1186/s12916-022-02697-3
22. Lee HJ, Manavalan A, Stefan-Lifshitz M, Schechter C, Maity A, Tomer Y. Permanent hypothyroidism following immune checkpoint inhibitors induced thyroiditis may be associated with improved survival: results of an exploratory study. Front Endocrinol (Lausanne). 2023 Apr 19;14:1169173. doi: 10.3389/fendo.2023.1169173.
23. Kotevski DP, Smee RI, Vajdic CM, Field M. Empirical comparison of routinely collected electronic health record data for head and neck cancer-specific survival in machine-learnt prognostic models. Head Neck. 2023 Feb;45(2):365-379. doi: 10.1002/hed.27241.
24. Wahli MN, Hayoz S, Hoch D, Ryser CO, Hoffmann M, Scherz A, Schwacha-Eipper B, Häfliger S, Wampfler J, Berger MD, Novak U, Özdemir BC. The role of immune checkpoint inhibitors in clinical practice: an analysis of the treatment patterns, survival and toxicity rates by sex. J Cancer Res Clin Oncol. 2023 Jul;149(7):3847-3858. doi: 10.1007/s00432-022-04309-2.
25. Okada T, Fushimi C, Matsuki T, Tokashiki K, Takahashi H, Okamoto I, Sato H, Kondo T, Hanyu K, Kishida T, Ito T, Yamashita G, Masubuchi T, Tada Y, Miura K, Omura GO, Yamashita T, Oridate N, Tsukahara K. Effects of Pembrolizumab in Recurrent/Metastatic Squamous Cell Head and Neck Carcinoma: A Multicenter Retrospective Study. Anticancer Res. 2023 Jun;43(6):2717-2724. doi: 10.21873/anticanres.16438.
26. Alnemri AE, Tekumalla S, Moroco AE, Vathiotis I, Tuluc M, Gargano S, Zhan T, Cognetti DM, Curry JM, Argiris A, Linnenbach A, South AP, Harshyne LA, Johnson JM, Luginbuhl AJ. Predictive capacity of immune-related adverse events and cytokine profiling in neoadjuvant immune checkpoint inhibitor trials for head and neck squamous cell carcinoma. Cancer Med. 2024 Jun;13(11):e7370. doi: 10.1002/cam4.7370
27. Ariizumi Y, Hanai N, Asakage T, Seto A, Tomioka T, Miyabe J, Kessoku H, Mukaigawa T, Omura G, Teshima M, Nishikawa D, Saito Y, Asada Y, Fujisawa T, Makino T, Nishino H, Sano D, Nakahira M, Tokashiki K, Uemura H, Ueda T, Sakai A, Masuda M, Tsujikawa T, Hiei Y, Nishio N, Matsui H, Kiyota N, Homma A. Extent of thyroidectomy and paratracheal lymph node dissection in total pharyngolaryngectomy for pyriform sinus cancer, and recurrence, survival, and postoperative hypoparathyroidism: A multicenter retrospective study. Head Neck. 2024 Feb;46(2):269-281. doi: 10.1002/hed.27572
28. Kennedy OJ, Ali N, Lee R, Monaghan P, Adam S, Cooksley T, Lorigan P. Thyroid dysfunction after immune checkpoint inhibitors in a single-centre UK pan-cancer cohort: A retrospective study. Eur J Cancer. 2024 May;202:113949. doi: 10.1016/j.ejca.2024.113949.
29. Thapa A, Cowell A, Peters A, Noble DJ, James A, Lamb C, Grose D, Vohra S, Schipani S, Mactier K, Mackenzie J, Srinivasan D, Laws K, Moleron R, Niblock P, Soh FY, Paterson C, Wilson C. The UK Divide: Does Having a Pembrolizumab-Chemotherapy Option in Head and Neck Cancer Matter? Real-world Experience of First-line Palliative Pembrolizumab Monotherapy and Pembrolizumab-Chemotherapy Combination in Scotland. Clin Oncol (R Coll Radiol). 2024 May;36(5):287-299. doi: 10.1016/j.clon.2024.02.004.

| **NEWCASTLE - OTTAWA SCALE** | | | | | | | | | | |
| --- | --- | --- | --- | --- | --- | --- | --- | --- | --- | --- |
|  |  | **Selection** | | | | **Comparability** | **Outcome** | | | **Overall Score** |
| **Sl.No** | **Study** | **Representativeness of the exposed cohort** | **Selection of the non-exposed cohort** | **Ascertainment of exposure** | **Demonstration that outcome of interest was not present at start of study** | **Comparability of cohorts on the basis of the design or analysis** | **Assessment of outcome** | **Was follow-up long enough for outcomes to occur** | **Adequacy of follow up of cohorts** |  |
| 1 | Nelson et al. 2006 Ohio, US | 1 | 1 | 1 | 1 | 1 | 1 | 1 | 0 | Score7/9 |
| 2 | Economopoulou et al. 2020 Greece | 1 | 1 | 1 | 1 | 2 | 1 | 1 | 1 | Score 9/9 |
| 3 | Jank et al. 2021 Austria | 1 | 1 | 1 | 1 | 2 | 1 | 1 | 1 | Score 9/9 |
| 4 | Weng et al. 2022 China | 1 | 1 | 1 | 1 | 2 | 1 | 1 | 1 | Score 9/9 |
| 5 | Haas et al. 2023 Vienna,Austria | 1 | 1 | 1 | 1 | 2 | 1 | 1 | 1 | Score-9/9 |
|  | **COCHRANE RoB2** | | | | | | | | | |
| **Sl.No** | **Study** | **Bias Arising from the Randomization Process** | **Bias Due to Deviations from Intended Interventions** | **Bias due to missing outcome data** | **Bias in Measurement of the Outcome** | **Bias in Selection of the Reported Result** | **Overall RoB2** |  |  |  |
| 1 | Patil et al. 2018 Mumbai, India | Low | Some concern | Low | Some concern | Low | Some concern |  |  |  |

**Appendix 4: Risk of bias assessment of the included studies**

Appendix5: Figure A


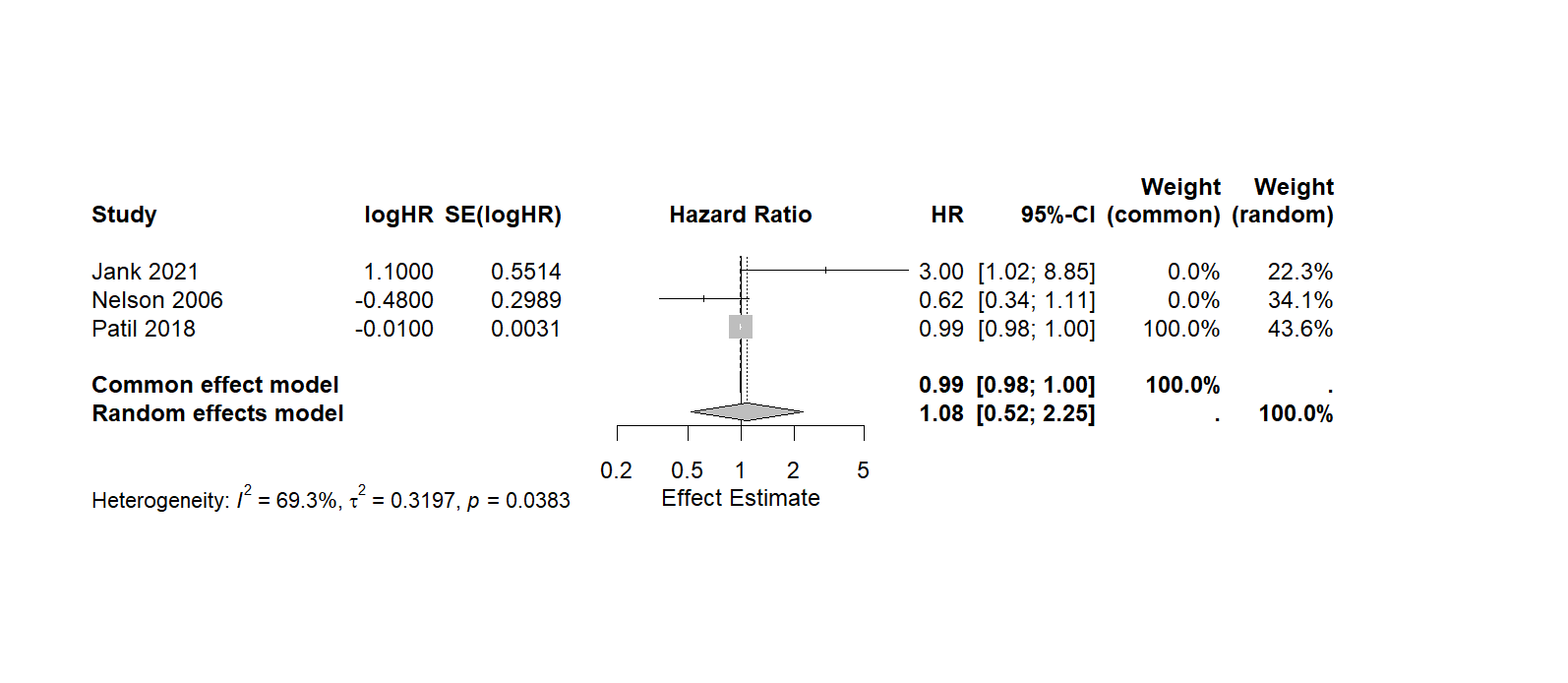


Figure A: Forest Plot of Meta-Analysis after excluding study conducted by Haas et al. (2023)
